# Segmentation of Older People’s Needs and Readiness for Smart Homes by Residentially Based Lifestyles in Spain: Survey Study

**DOI:** 10.2196/75110

**Published:** 2026-05-05

**Authors:** Jiyeon Yu, Angélica de Antonio, Elena Villalba-Mora

**Affiliations:** 1 Centre for Biomedical Technology, Universidad Politécnica de Madrid, Parque Científico y Tecnológico de la UPM, Campus de Montegancedo, Crta. M40, Km. 38, Pozuelo de Alarcón, Madrid, 28223, Spain, 34 619490028

**Keywords:** older people, smart homes, assistive technology, user needs, user segmentation, residentially based lifestyles

## Abstract

**Background:**

Globally, the older population is increasing rapidly, becoming one of the most significant demographic trends of the 21st century. This growth poses important social, health, and technological challenges for societies that must adapt their environments and services to promote independent and healthy aging. In Spain, the population aged 65 years and older reached 18% of the total population in 2020, and projections indicate that this proportion will continue to rise in the coming decades. Within this context, smart homes have emerged as one of the most promising avenues to support aging in place and improve the quality of life. Smart homes encompass a wide variety of functions, including environmental control, safety monitoring, communication, and other assistive technologies, that may help older people stay healthy, safe, and independent in their own homes. However, older people are not a homogeneous group. Their lifestyles, health conditions, and technological experiences differ substantially, which means that, as with any assistive technology, smart home functions must match the real and perceived needs of the target users to ensure acceptance, adoption, and long-term use.

**Objective:**

In this study, as a step forward toward the adaptability of smart home technology, we present a method to analyze the practical needs of smart home functions for older people. Specifically, we aim to understand the Spanish older population’s readiness and needs for smart homes and to provide insights that can guide the design of more adaptive and user-centered solutions.

**Methods:**

We conducted an online survey focusing on residentially based lifestyles, health conditions, and preferences for smart home functions, targeting older adults living in Spain. The survey collected information about participants’ demographic profiles, daily activities, health self-assessment, and attitudes toward technology. A total of 102 valid responses were analyzed. We then classified the older adults according to their residentially based lifestyles using clustering techniques and analyzed the preferences and needs for smart home functions in each identified group.

**Results:**

Four clusters emerged based on the information provided by the participants: (1) high quality of life and independent life, (2) poor quality of life, (3) social-centered life, and (4) creative and personal-centered hobbies at home. On the basis of this classification, we explored each group’s specific needs for smart homes and estimated their readiness to embrace different aspects of technology. As a result, the top-priority smart home functions for each group were identified and compared.

**Conclusions:**

This research contributes to understanding the practical user needs of smart homes as assistive technologies for older people. It provides a methodological approach to anticipate and prioritize functions according to user characteristics, supporting the development of personalized, adaptive, and more acceptable smart home solutions for aging populations.

## Introduction

Population aging is a global challenge [1]. It is of utmost importance for older people to stay healthy and independent in their homes, where they feel comfortable. To support independent living, technology that can quickly detect hazards and respond appropriately in emergency situations becomes crucial for older people living in their own homes [2].

Smart home technology has made significant advances in the fields of remote management and older adult assistance [3]. Smart home services improve comfort, safety, and security at home with Internet-of-Things technology and allow older adults to receive medical treatment or check their health at home [4]. It is becoming a very important technology to support healthy and independent lives for older people [5]. Ultimately, this technology can help improve the quality of life in the older population [6].

However, most older adults perceive smart technologies to be difficult to use, and there may be many barriers to adopting the technology in real life. In addition, older people may not even be aware of how smart home functions can meet their needs. For these reasons, it is expected that commercialization and widespread use of smart homes for older adults will take still some time [6]. In order for this technology to pervade their lives, older users should not be classified into a single group but should be subdivided according to the service purpose, so their specific needs can be identified and satisfied accordingly [7].

To address this challenge, we segmented older people according to their lifestyles within residential facilities, where smart homes are used. Our segmentation was based on residentially based lifestyles (RBL). Some studies claim that lifestyles related to a specific domain can provide meaningful results for segmentation of the population under study [8]. For example, Thøgersen [9] considered food-related lifestyles for analysis related to food consumption, and Sanquist et al [10] proposed the use of energy-related lifestyles as a domain to identify patterns of energy consumption.

In this study, we used a questionnaire on RBL and smart home needs that we have previously developed and validated in a Korean population, in which we confirmed that this is a tool that can lead to meaningful results [11]. Building upon that prior work, the present research applies the instrument to a sample of older adults in Spain, with the aim of proposing a segmentation method and analyzing the smart home requirements of each subgroup. This approach enables the assessment of both readiness and specific needs, thereby generating insights that can inform strategies to enhance the adoption and usability of smart home technologies among older adults.

The remainder of the paper is organized as follows. The *Methods* section presents the study process, the participants, the questionnaire used, and the data analysis. Then, the *Results* section presents the principal component analysis (PCA) of the RBL, the segmentation, and the needs for the smart home functions. The *Discussion* section states first the main findings, comparing them with related works, and then it discusses the limitations. Finally, the *Conclusions* section closes the paper with the main contribution and future research.

## Methods

### Process

**Figure 1.**
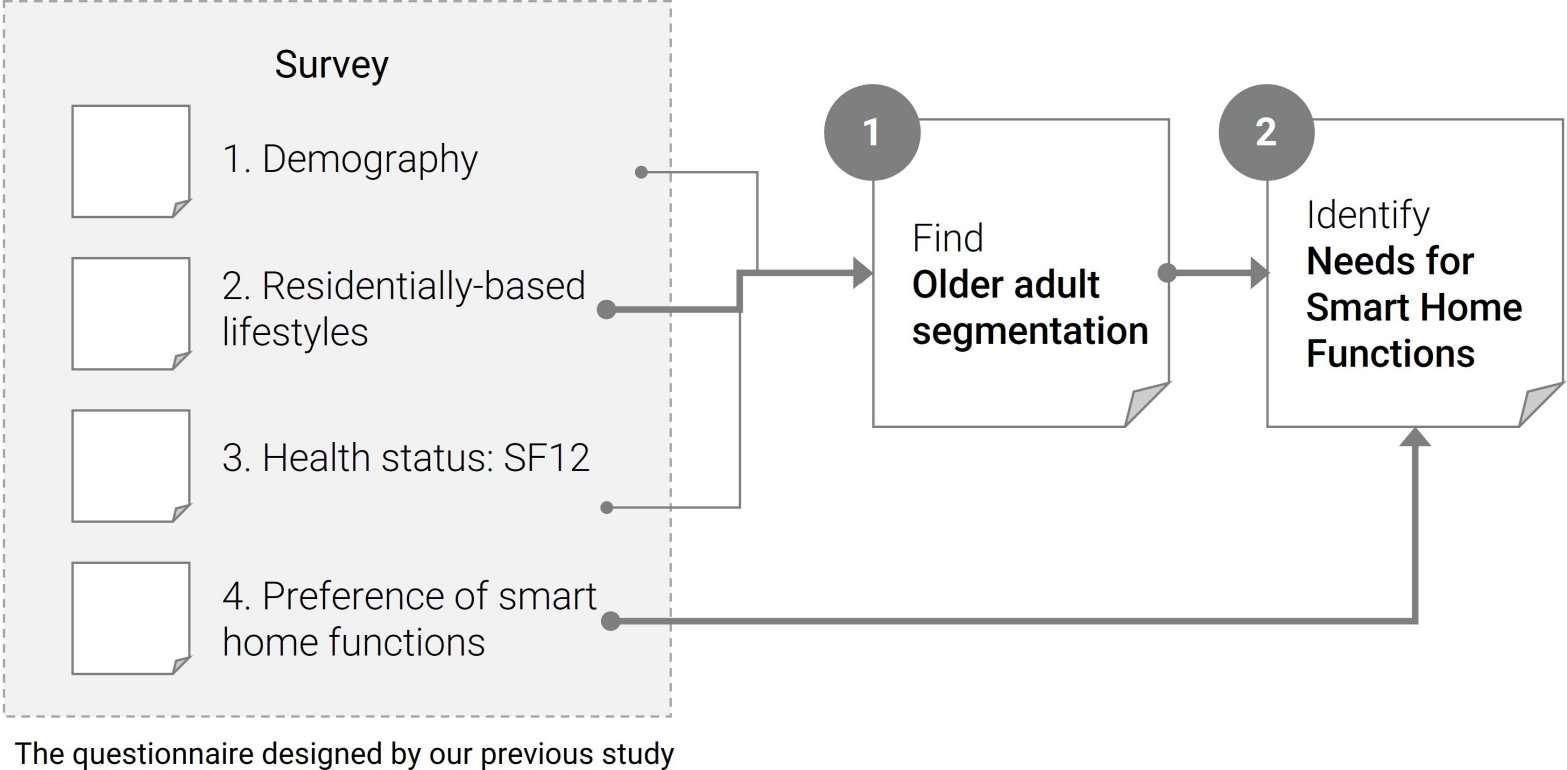
Study process. SF12: 12-item Short-Form Health Survey.

Figure 1 presents the study process. The resulting outcomes are (1) RBL-based segmentation of older adults in Spain and (2) analysis of the needs of smart home functions for each segment. This study proves that segmentation according to RBL and analysis of smart home needs through the questionnaire and method presented are useful to analyze the perception and detailed needs of smart homes, which are essential as assistive technologies for older adults.

### Participants

This study targeted older adults in Spain, a representative country facing an aging society. In the previous Korean study, the survey was conducted with older adults aged 65 years or older. Considering that the internet usage of older adults in South Korea decreases at higher ages than in Spain [12,13], we set the lower age limit at 60 years, 5 years below that of South Korea.

**Figure 2.**
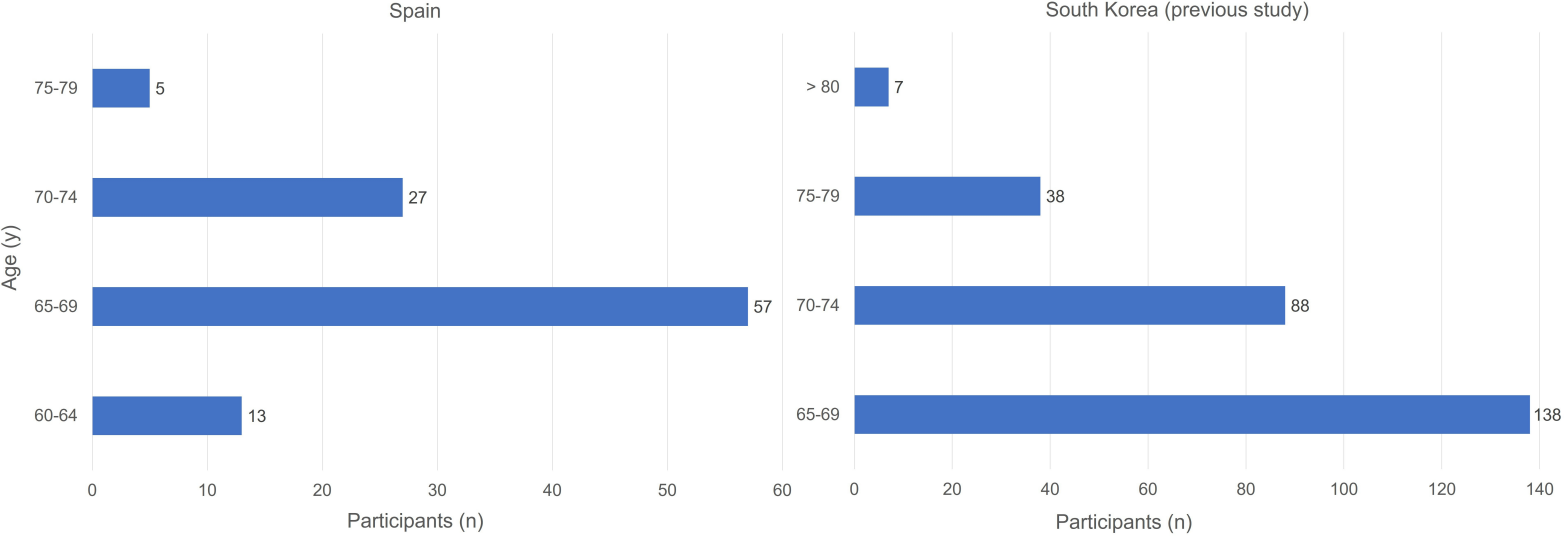
Age comparison of participants in 2 countries.

Therefore, the survey participants were designated as senior citizens aged 60 years and over, living in Spain (Figure 2). The criteria for a valid response were (1) age of 60 years or older, (2) no missing answers to questions, and (3) responses received within the specified survey period. We collected a total of 102 valid answers over 5 consecutive weeks. Regarding the proportion of women and men, our sample included 41 men and 61 women (40.2% vs 59.8%), similar to that of Spain (45% vs 55%) [14]. The average age of the respondents was 68 (SD 3.8) years.

### Instruments

We used a questionnaire that was developed and used in our previous Korean study [11], which consisted of a total of four sections: (1) demographics, (2) RBL, (3) health status, and (4) smart home function preferences. The demographics section consisted of eight questions: gender, age, marital status, residence type, caregiver, time spent out of the home, education, and monthly income. Questions for RBL were constructed by analyzing existing relevant studies [15-23] and explored the following: (1) quality of home life and needs for daily life, (2) daily home activities, and (3) social events at home.

In addition, the health status was checked through the Short-Form Health Survey of 12 items, so that the physical and mental health of each group could be compared after segmentation by RBL questions. With proven reliability and validity, the 12-item Short-Form Health Survey was developed to measure the health status of respondents in a short time. This survey calculates a physical component summary (PCS) and a mental component summary (MCS), which can be assessed based on the US average of 50 points. A high score means good status of physical or mental health [24].

Finally, regarding smart home function preferences, functions considered were based on a systematic review of smart home research [25]. On the basis of the results of this study, we could extract 26 functions in 8 categories. This section of the questionnaire demands the participant to select from 1 (not necessary) to 5 (most necessary) how much each of the 26 smart home functions is needed.

All questionnaires were carefully revised by experts in gerontology research and smart home technology.

### Procedure

Data collection was conducted as an online survey, a non-face-to-face survey tool. This survey used convenience sampling, which is one of the nonprobability sampling methods. The questionnaire was distributed among older adults in different areas of the Madrid region via day care centers. The questionnaire was translated into Spanish, and it was distributed via Google Forms, together with a guide on the purpose of the study and the information that the survey responses would not be used for any other purpose except for research. Responses were collected over 5 weeks.

### Ethical Considerations

We did not gather any personal data that could be linked in any way to the identity of the respondents; therefore, according to the rules of our ethical committee at Universidad Politécnica de Madrid [26], there was no need to request the prior approval of the committee. The survey does not require any personally identifiable data (eg, name, phone, email, and personal address) and does not require going through a log-in process in which personal information may be collected. In addition, respondents could voluntarily choose whether to participate in the survey or not; therefore, starting the survey constitutes a valid consent to participate. The collected responses were stored and analyzed anonymously. Moreover, the collected answers were not used for any purpose other than research analysis, and all initial data from the survey were deleted after analysis. No interventions, compensation, or treatments were included in this research.

### Data Analysis

The items on RBL were dimensionally reduced to main factors through PCA. By applying PCA before clustering, the original 17 survey items were reduced to five interpretable dimensions: (1) independent living and quality of life, (2) social events at home, (3) regular life routine, (4) creative and healthy, and (5) time spent alone. This dimensional reduction served 2 main purposes. First, it minimized noise by removing items with low communalities and condensed correlated items into coherent factors, thereby improving the stability of the clustering solution. Second, it enhanced interpretability, as clusters could be explained in terms of meaningful lifestyle dimensions rather than raw questionnaire items. In high-dimensional spaces (many variables), distance metrics become less discriminating, and clustering algorithms such as K-means can perform poorly or yield unstable clusters. Compared to direct clustering on the original 17 variables, clustering on the 5 extracted components provided clearer group separation and yielded more distinct, interpretable lifestyle-based clusters. In contrast, clusters formed directly on all 17 items would be harder to interpret because each cluster’s distinguishing features would involve possibly many items with varying contributions. Moreover, parsimony in model building (ie, fewer variables) tends to generalize better when replicating or applying to new data. It is important to highlight that PCA does sacrifice some information present in the original variables, especially if lower variance components (or items with unique variance) are dropped; some cluster-defining features might lie in those discarded dimensions. In addition, PCA assumes linear relationships among items and may not capture complex nonlinear interactions.

For reliability analysis of the resulting factors, internal consistency was verified with Cronbach α value. K-means clustering based on these RBL factors could segment Spanish older respondents with similar patterns of RBL and characteristics. After segmentation by RBL, the demographics and health status of each group were compared. Then, through the questions on smart home function preferences, the needs for smart home functions for each group were measured.

To verify significant differences among groups, chi-square test validation was applied to the demographic data, and ANOVA analysis was used on the health status scores (PCS and MCS) and smart home needs scale.

## Results

### PCA of RBL

The mean Kaiser-Meyer-Olkin value for the RBL items was 0.604. After removing factors with commonalities of low scores, PCA was performed. Bartlett’s test of sphericity was significant (*χ*²_136_=455.893; *P*<.001), indicating that the correlation matrix was not an identity matrix and was therefore suitable for factor analysis. PCA was selected to reduce the dimension to the main factors of each assessment item and analyzed by Varimax rotation. Table 1 presents the PCA results of the RBL items. Five principal factors were extracted by allowing factor loadings greater than 0.4: (1) independent living and quality of life, (2) social event at home, (3) regular life routine, (4) creative and healthy, and (5) time spent alone.

**Table 1.** Principal component analysis on residentially based lifestyles (RBL questions [11]).

RBL question	Independent living and quality of life	Social event at home	Regular life routine	Creative and healthy	Time spent alone
Independent living and quality of life (factor loadings)					
My chronic disease doesn't affect the quality of my daily life.	0.828	—^a^	—	—	—
My medication or treatments doesn't affect the quality of my daily life.	0.827	—	—	—	—
Generally, I feel satisfied with my daily life.	0.718	—	—	—	—
I don't need help to clean the house, wash the dishes or do the laundry.	0.695	—	—	—	—
I am satisfied with how I share my time.	0.599	—	—	—	—
I eat at least 3 times a day.	0.415	—	—	—	—
Social event at home (factor loadings)					
Invite friends	—	0.788	—	—	—
Visits of another type	—	0.784	—	—	—
Family visit	—	0.636	—	—	—
Regular life routine (factor loadings)					
My diet is varied and balanced.	—	—	0.732	—	—
I am not sleepy during the day.	—	—	0.707	—	—
Take care of my house	—	—	0.489	—	—
Creative and healthy (factor loadings)					
Cook or bake	—	—	—	0.732	—
Creative and artistic activities	—	—	—	0.646	—
Do light exercise	—	—	—	0.539	—
Time spent alone (factor loadings)					
Use a computer or smartphone or tablet at home	—	—	—	—	0.775
Read books, newspapers or magazines	—	—	—	—	0.626
Sum of squares loadings	3.051	2.034	1.674	1.625	1.593
Proportion variance	17.949	11.965	9.847	9.56	9.372
Cumulative variance	17.949	29.914	39.761	49.32	58.692
Cronbach α	0.8	0.651	0.489	0.424	0.449

a Not applicable.

The reliability of each factor was confirmed by the Cronbach α value. Although Cronbach α values of 3 factors were measured somewhat lower, those were accepted considering that the questionnaire was newly developed in the previous study and the survey was conducted with a limited number of people. Each factor has at least an eigenvalue of 1, and the total variance explained by these 5 factors is 58.69%. According to methodological studies in the social sciences, cumulative explained variance values of approximately 50% to 60% are considered adequate for survey-based instruments.

### Segmentation Based on RBL

**Figure 3.**
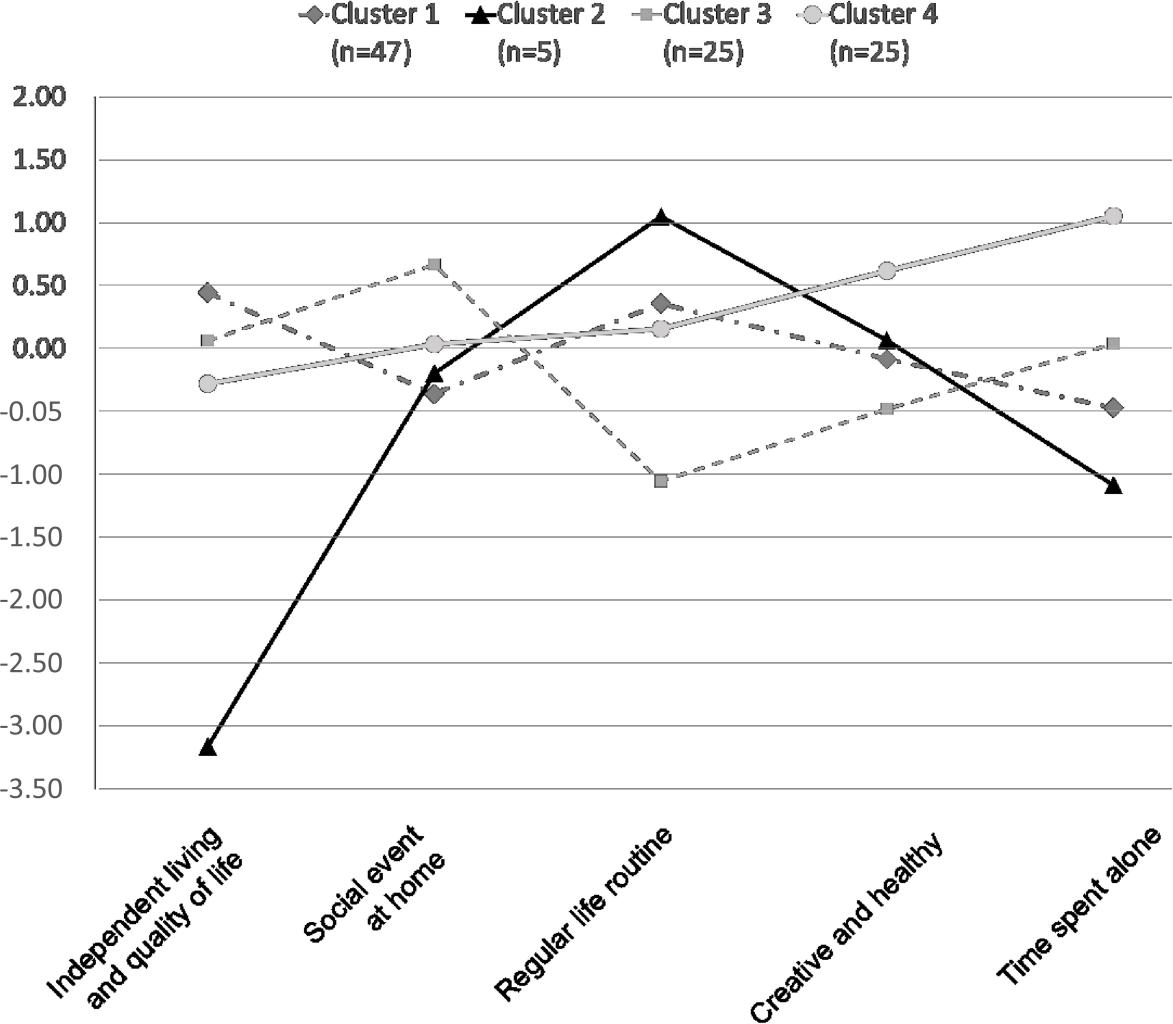
Clusters for older people by the residentially based lifestyles.

On the basis of 5 RBL factors, a hierarchical cluster analysis via the Ward method was conducted, and it was judged that classification into 4 clusters was the most stable and convincing. After that, final clustering was performed through K-mean cluster analysis. Table 2 presents the clustering results segmented into the 4 groups. ANOVA analysis was conducted to explore differences in RBL factors by clusters. The analysis shows that all factors are significantly different: (1) independent living and quality of life (50.80; *P*<.001), (2) social event at home (6.83; *P*<.01), (3) regular life routine (21.29; *P*<.001), (4) creative and healthy (5.98; *P*<.01), and (5) time spent alone (25.43; *P*<.001). Figure 3 shows the differences among the factors for each cluster.

The lifestyle patterns and characteristics of each group were defined according to the 5 factor values in the RBL.

- Cluster 1: “High quality and independent life” is a group that shows the highest quality of life and independent living ability.
- Cluster 2: “Poor quality of life” has the lowest quality of life compared to other groups and the least amount of time spent on hobbies at home alone. It also displays the most regular life routine.
- Cluster 3: “Social-centered life” has a lifestyle with the most frequent visits by friends and family to their homes and the least regular life routine.
- Cluster 4: “Creative and personal-centered hobbies at home” is a group of people who have many creative hobbies and activities that they can do alone.

**Table 2.** Cluster analysis for residentially based lifestyles (RBL) of older people.

	Cluster 1 (n=47), factor scores (mean)	Cluster 2 (n=5), factor scores (mean)	Cluster 3 (n=25), factor scores (mean)	Cluster 4 (n=25), factor scores (mean)	*F* test (*df*)
Independent living and quality of life	0.45	−3.16	0.07	−0.28	50.80^a^ (3, 98)
Social event at home	−0.36	−0.19	0.67	0.04	6.83^a^ (3, 98)
Regular life routine	0.36	1.05	−1.05	0.16	21.29^a^ (3, 98)
Creative and healthy	−0.08	0.07	−0.48	0.62	5.98^b^ (3, 98)
Time spent alone	−0.47	−1.08	0.04	1.06	25.43^a^ (3, 98)

a *P*<.001.

b .001≤*P*<.01*.*

Table 3 presents the demographic characteristics of each group. There is a significant difference in gender of each group. Clusters 1 and 4 had more females with a male-to-female ratio of approximately 1:2, and cluster 2 is made up of 100% females. Cluster 3 was the only group in which the proportion of men was higher.

The MCS score of cluster 1 “high quality and independent life” was the highest, and the PCS score of cluster 4 “creative and personal-centered hobbies at home” is the highest. On the other hand, cluster 2 “poor quality of life” had the lowest both PCS and MCS ().

A one-way analysis of variance was conducted to examine differences in health status across the four residentially based lifestyle clusters. The results indicated statistically significant differences in physical component scores among the clusters (*P*=.01). The mean physical component scores were 50.33 for Cluster 1 (high quality of life and independent), 25.73 for Cluster 2 (poor quality of life), 47.74 for Cluster 3 (social-centered life), and 50.45 for Cluster 4 (creative and personal-centered hobbies at home). Specifically, Cluster 2 demonstrated substantially lower physical component scores compared with the other clusters, whereas Clusters 1, 3, and 4 showed relatively higher and comparable scores.

In contrast, differences in mental component scores did not reach statistical significance (*P*=.15). The mean mental component scores were 52.88 (SD 8.42) for Cluster 1, 43.48 (SD 10.12) for Cluster 2, 52.87 (SD 7.95) for Cluster 3, and 51.66 (SD 9.03) for Cluster 4, suggesting that mental health status was relatively similar across clusters.

**Table 3.** Demographics of each cluster.

	Cluster 1 (high quality of life and independent life; n=47), n (%)^a^	Cluster 2 (poor quality of life; n=5), n (%)^b^	Cluster 3 (social-centered life; n=25), n (%)^c^	Cluster 4 (creative and personal-centered hobbies at home; n=25), n (%)^d^	Chi-square (*df*)
Gender					12.9 (3)^e^
Male	15 (31.9)	0 (0.0)	17 (68.0)	9 (36.0)	
Female	32 (68.1)	5 (100.0)	8 (32.0)	16 (64.0)	
Age (y)					6.6 (9)
60-64	6 (12.8)	0 (0)	4 (16.0)	3 (12.0)	
65-69	26 (55.3)	4 (80.0)	13 (52.0)	14 (56.0)	
70-74	14 (29.8)	0 (0.0)	6 (24.0)	7 (28.0)	
75-79	1 (2.1)	1 (20.0)	2 (8.0)	1 (4.0)	
Marital status					16.3 (9)
Married	32 (68.1)	2 (40.0)	18 (72.0)	13 (52.0)	
Single	3 (6.4)	2 (40.0)	5 (20.0)	2 (8.0)	
Widowed	3 (6.4)	1 (20.0)	2 (8.0)	4 (16.0)	
Separated	9 (19.1)	0 (0)	0 (0.0)	6 (24.0)	
Resident type					11.9 (9)
Alone	12 (25.5)	2 (40.0)	6 (24.0)	11 (44.0)	
With spouse	30 (63.8)	2 (40.0)	14 (56.0)	10 (40.0)	
With family	4 (8.5)	0 (0)	4 (16.0)	4 (16.0)	
Others	1 (2.1)	1 (20.0)	1 (4.0)	0 (0)	
Caregiver					12.2 (12)
Spouse	33 (70.2)	2 (40.0)	18 (72.0)	13 (52.0)	
Son or daughter	3 (6.4)	1 (20.0)	2 (8.0)	5 (20.0)	
Other family	1 (2.1)	0 (0)	3 (12.0)	1 (4.0)	
Friends or neighbor	5 (10.6)	1 (20.0)	1 (4.0)	2 (8.0)	
Others	5 (10.6)	1 (20.0)	1 (4.0)	4 (16.0)	
Spent time out of home (h)					10.5 (9)
<1	2 (4.3)	2 (40.0)	2 (8.0)	4 (16.0)	
1-3	23 (48.9)	3 (60.0)	12 (48.0)	10 (40.0)	
3-6	18 (38.3)	0 (0.0)	8 (32.0)	8 (32.0)	
>6	4 (8.5)	0 (0.0)	3 (12.0)	3 (12.0)	
Education					8.3 (6)
Basic	1 (2.1)	1 (20.0)	2 (8.0)	0 (0)	
Intermediate	8 (17.0)	2 (40.0)	4 (16.0)	4 (16.0)	
University	38 (80.9)	2 (40.0)	19 (76.0)	21 (84.0)	
Income (€)^f^					5.5 (9)
<1500	5 (10.6)	1 (20.0)	2 (8.0)	2 (8.0)	
1500-2000	7 (14.9)	1 (20.0)	3 (12.0)	4 (16.0)	
2001-2999	15 (31.9)	3 (60.0)	7 (28.0)	10 (40.0)	
>3000	20 (42.6)	0 (0)	13 (52.0)	9 (36.0)	

a Residentially based lifestyles: They have independent life, high quality of life, and low social activity at home.

b Residentially based lifestyles: They have low quality of life and less personal free time.

c Residentially based lifestyles: They have many social events and low time spent alone.

d Residentially based lifestyles: They spend a lot of time at home and do various activities alone.

e .001≤*P*<.01*.*

f €1=US $1.18.

### Needs for Smart Home Functions

In this section, we compared the needs for smart home functions of each cluster. The respondents could choose from 1 (not necessary) to 5 (most necessary) for 26 detailed smart home functions in 8 categories. Higher numbers should be interpreted to mean “more necessary functions” for their lives (Table 4). As a result of ANOVA, there was a significant difference in the preference—F(3, 98)—of the *sensors you can wear as clothes* function. The smart home needs of cluster 2 “poor quality of life” are the highest, with an average score of 2.06 for a total of 26 functions, while the needs of cluster 1 “high quality of life and independent life” are the lowest, with an average score of 1.75.

We compared the needs of smart home functions above the median, based on the Spanish responses (value=2) (highlighted in italic in Table 4 and ordered by decreasing priority in Table 5). Cluster 2 “poor quality of life” needed more smart home functions compared to other groups. On the other hand, “high quality and independent life” and “social-centered life” had relatively few needs for the smart home functions.

There was also difference in the most essential functions for each group. Cluster 1 “high quality and independent life” required the *gas leak detection or smoke detection* function the most, cluster 2 “poor quality of life” required the *telehealthcare* function, cluster 3 “social-centered life” required the *remote-controlled electricity* function, and cluster 4 “creative and personal-centered hobbies at home” required the *automatic heating* function the most.

However, it is important to highlight that there are three functions*—automatic heating*, *telehealthcare*, and *call the family in case of emergency—*which are essential in all 4 groups. This indicates that the Spanish older population is ready to adopt such services. On the other hand, we observed the functions that were not considered important in any of the groups: *automatic watering in the garden*, *automatically checked postbox*, *monitoring in your absence*, and *robot like a friend*. Table 5 lists which smart home functions are needed according to the perceived priority in each group.

**Table 4.** The needs for smart home functions (function list [11]).

Smart home functions	Cluster 1 (n=47, 46.1%; high quality of life and independent life), mean (SD)	Cluster 2 (n=5, 4.9%; poor quality of life), mean (SD)	Cluster 3 (n=25, 24.5%; social-centered life), mean (SD)	Cluster 4 (n=25, 24.5%; creative and personal-centered hobbies at home), mean (SD)	*F* test (*df*)
Smart washing machine	1.85 (0.83)	*2.2*^a^ *(1.10)*	1.84 (0.85)	1.8 (0.76)	0.045 (3, 98)
Smart refrigerator	1.45 (0.65)	*2.2 (0.84)*	1.6 (0.71)	1.76 (0.78)	1.374 (3, 98)
Smart cooker and smart coffee maker	1.43 (0.62)	*2.4 (1.14)*	1.72 (0.79)	1.76 (0.83)	2.468 (3, 98)
Schedule reminder	1.49 (0.66)	*2.4 (1.14)*	1.44 (0.65)	1.76 (0.83)	0.673 (3, 98)
Reminder to take medicine	*1.55 (0.71)*	*2.6 (1.34)*	1.32 (0.56)	1.52 (0.71)	0.409 (3, 98)
Open windows or doors detection	1.62 (0.74)	*2 (1.00)*	1.48 (0.65)	1.64 (0.76)	0.035 (3, 98)
Automatic watering in the garden	1.77 (0.84)	1 (0)	1.52 (0.71)	1.96 (0.93)	0.128 (3, 98)
Automatically checked postbox	1.72 (0.80)	1 (0)	1.68 (0.75)	1.88 (0.88)	0.366 (3, 98)
Automatic heating	*2.28 (1.08)*	*2.8 (1.30)*	*2.24 (1.05)*	*2.52 (1.20)*	0.359 (3, 98)
Gas leak detection or smoke detection	*2.38 (1.12)*	*1.8 (0.84)*	*2.12 (1.01)*	*2.48 (1.16)*	0 (3, 98)
Remote-controlled electricity	*2.06 (0.95)*	*1.4 (0.55)*	*2.28 (1.08)*	*2.4 (1.12)*	1.93 (3, 98)
Remote-controlled lights	1.77 (0.84)	1.8 (0.84)	1.8 (0.82)	*2.08 (0.95)*	1.269 (3, 98)
Light mode settings	1.77 (0.84)	1.8 (0.84)	1.88 (0.88)	*2.24 (1.05)*	3.23 (3, 98)
TV auto-play function, notifications from my channel	1.66 (0.77)	2.4 (1.14)	1.72 (0.79)	1.88 (0.88)	0.779 (3, 98)
Personalized learning TV content	1.79 (0.86)	1.6 (0.55)	1.44 (0.65)	*2 (1.00)*	0.072 (3, 98)
Automatic cinema mode setting	1.72 (0.80)	2 (1.00)	1.8 (0.82)	1.76 (0.78)	0.053 (3, 98)
Fall detection	*2.23 (1.08)*	*2.8 (1.30)*	1.92 (0.91)	2.00 (1)	0.985 (3, 98)
Monitoring in your absence	1.74 (0.81)	1.8 (0.84)	1.56 (0.71)	1.96 (0.94)	0.216 (3, 98)
Measure sleep health	1.77 (0.84)	1.8 (0.84)	*2 (1)*	*2.04 (1.02)*	1.77 (3, 98)
Sensors you can wear as clothes	1.53 (0.70)	*2.2 (1.1)*	1.88 (0.88)	1.96 (0.94)	3.965^b^ (3, 98)
Mental health detection	1.47 (0.66)	*2.4 (1.14)*	1.84 (0.85)	1.84 (0.85)	3.035 (3, 98)
Telehealthcare	*2.06 (1)*	*3 (1.41)*	*2.16 (1.03)*	*2.4 (1.12)*	0.002 (3, 98)
Home management assistant robot	1.6 (0.75)	*2.4 (1.14)*	*2 (1)*	1.76 (0.83)	1.115 (3, 98)
Equipment to help you up and down stairs	1.34 (0.57)	*2 (1)*	1.2 (0.41)	1.68 (0.79)	1.313 (3, 98)
Robot like a friend	1.28 (0.54)	1.2 (0.45)	1.28 (0.54)	1.32 (0.56)	0.073 (3, 98)
Call the family in case of emergency	*2.13 (1.02)*	*2.6 (1.34)*	*2.12 (1.01)*	*2.04 (1.02)*	0.098 (3, 98)
Average of needs	1.75	*2.06*	1.76	1.94	—^c^

a Italics indicate values higher than the median of 2.0.

b Statistical significance at the *P*<.05 level.

c Not applicable.

**Table 5.** Smart home function priority for each cluster.

Priority	Cluster 1 (n=47, 46.1%; high quality of life and independent life)	Cluster 2 (n=5, 4.9%; poor quality of life)	Cluster 3 (n=25, 24.5%; social-centered life)	Cluster 4 (n=25, 24.5%; creative and personal-centered hobbies at home)
1	Gas leak detection or smoke detection	Telehealthcare	Remote-controlled electricity	Automatic heating
2	Automatic heating	Automatic heating and fall detection	Automatic heating	Gas leak detection or smoke detection
3	Fall detection	Reminder to take medicine and call the family in case of emergency	Telehealthcare	Remote-controlled electricity and telehealthcare
4	Call the family in case of emergency	Smart cooker and smart coffee maker, schedule reminder, TV auto-play function, notifications from my channel, mental health detection, and home management assistant robot	Gas leak detection or smoke detection and call the family in case of emergency	Light mode settings
5	Remote-controlled electricity	Smart washing machine	Measure sleep health	Remote-controlled lights

## Discussion

### Principal Findings

This study identifies the smart home needs of older adults in Spain through a segmentation according to RBL. Through this approach, we can assess the readiness of older people to use and adopt concrete smart home functions. Concretely, we found 4 clusters of the Spanish older adults, identified the smart home functions that each group considers important, and analyzed how the smart home needs of older users relate to RBL.

To derive meaningful lifestyle clusters from the original 17 survey items, we first applied PCA to reduce dimensionality before performing K-means clustering on the extracted component scores. Specifically, after removing items with low communalities, PCA with Varimax rotation yielded 5 interpretable components (ie, independent living and quality of life, social events at home, regular life routine, creative and healthy, and time spent alone), each having an eigenvalue of 1 or greater. These 5 components explained approximately 58.7% of the total variance in the data.

Using these components for clustering rather than the full set of original variables offers several methodological advantages:

1. Reduction of redundancy and noise: Some of the 17 items are correlated and share underlying variance; PCA consolidates that shared variance and filters out items or variance that contribute less to the common structure. This reduces noise (ie, irrelevant or weakly contributing variation) that might obscure true cluster structure.
2. Mitigation of the curse of dimensionality: In high-dimensional space (many variables), distance metrics become less discriminating, and clustering algorithms such as K-means can perform poorly or yield unstable clusters. By reducing dimensions to those that capture the majority of variance and represent interpretable dimensions, we improve the cluster algorithm’s ability to separate groups meaningfully.
3. Improved interpretability: Clusters defined on principal component scores are easier to characterize: each component is labeled (eg, “regular routine,” “social at home,” etc), so the resulting clusters can be described in terms of these meaningful dimensions. In contrast, clusters formed directly on all 17 items would be harder to interpret because each cluster’s distinguishing features would involve possibly many items with varying contributions.
4. Stability and parsimony: Working with fewer, more stable dimensions tends to improve the reliability of clustering; clusters are less likely to be driven by measurement error or idiosyncratic items. Also, parsimony in model building (ie, fewer variables) tends to generalize better when replicating or applying to new data.
5. Facilitation of validity checks: Components allow checking internal consistency (Cronbach α) for each extracted dimension, which helps ensure that the reduced dimensions are coherent constructs. Additionally, when the components are reliably measured, using them in clustering gives more confidence in subsequently interpreting differences among clusters.

In our case, after PCA, we used K-means clustering on the 5 component scores and identified 4 clusters with distinct lifestyle profiles. Compared to what clustering on the full 17 variables would likely have yielded, the component-based clustering produced more coherent, stable, and interpretable clusters. For example, rather than clusters being defined by numerous item-specific idiosyncrasies, each cluster’s profile can be described in terms of high or low scores on the 5 factors.

It should be noted that PCA does sacrifice some information present in the original variables, especially if lower variance components (or items with unique variance) are dropped; some cluster-defining features might lie in those discarded dimensions. Also, PCA assumes linear relationships among items and may not capture complex nonlinear interactions. Nonetheless, given our sample size and the moderate Kaiser-Meyer-Olkin (~0.60), the approach of PCA followed by K-means was judged appropriate.

Visutsak and Daoudi [27] attempted to identify the perceptions, accessibility factors, and needs of the older adults for smart homes through a survey. They asked about 7 smart home features in total and received responses from 20 stakeholders (doctors, nurses, the older adults, and their adult children). In a study on perception of smart home technologies [4], a survey was conducted with the general population, including working people in hospitals and nursing homes, to identify awareness of 6 smart home technologies for the older adults and concerns for using. This identified perceptions of usefulness for key technologies and their concerns from 18 respondents. Unlike previous studies, our study focuses on the older adults who will be the actual primary users. In addition, our questionnaire asked about the needs and perceptions of 26 detailed functions in a total of 8 categories of smart homes, covering a wider range of functions than previous studies. In addition, we compared and analyzed the particular needs of each group by segmentation based on the RBL.

In this study, rather than targeting older adults with a specific status (cognitive level and health status), the participants were segmented according to RBL. In addition, after segmentation, the average physical and mental health status of each group could be analyzed in connection with smart home needs. Although smart home technology could have great benefits for older people who need physical assistance or have mild cognitive impairment, we believe that smart home technology will help maintain independent living both for the healthy older adults and those with poor health.

Four segmented groups were identified in Spain: (1) high quality of life and independent life, (2) poor quality of life, (3) social-centered life, and (4) creative and personal-centered hobbies at home. We figured out the priority of smart home functions for each group and found that the priority of each group and the number of required functions were different. Although the 4 clusters provided meaningful and interpretable lifestyle profiles, 1 cluster (cluster 2, n=5) was substantially smaller than the others. At first glance, such a small subgroup may raise concerns about representativeness and the robustness of comparisons across clusters. However, several factors justify retaining this cluster in the analysis. First, the emergence of a small cluster was not an artifact of the analytic procedure but rather a reflection of genuine heterogeneity within the data. Even after multiple reclusterings, cluster 2 consistently appeared, suggesting that it represents a stable and reproducible pattern rather than a random byproduct of the algorithm. Second, the value of clustering lies not only in identifying large, dominant groups but also in revealing smaller, distinctive subgroups. In many areas of social science and consumer research, rare or niche groups play a crucial role in theory development and practice, despite their limited size [28,29]. Third, prior work in clustering methodology emphasizes that the interpretability and stability of clusters are more critical than their absolute size [30]. From this perspective, the consistent detection of cluster 2 across replications strengthens its validity as a meaningful subgroup. Finally, while the small number of respondents in this cluster warrants caution in generalization, its unique profile provides important insight into lifestyle heterogeneity. Future research with larger and more diverse samples can further verify the existence and characteristics of this subgroup, but its inclusion in this study enhances rather than detracts from the comprehensiveness of the clustering solution.

Cluster 2 “poor quality of life,” which is the group with the weakest mental and physical health, required more smart home functions than the other clusters. Their top priority is *Telehealth*, a system that allows them to receive medical care without going to a hospital. Besides that, they valued almost all categories of features as important; especially, they considered the *automation of daily routines* category and the *assist mobility* category as more important than the other groups. Also, as they spend more time at home than other groups, it was confirmed that they were interested in setting up TV programs and movie modes to enjoy at home.

Although only a limited number of functions showed statistically significant differences across the 4 clusters, this does not necessarily diminish the value of the segmentation. Clustering methods aim primarily to uncover underlying heterogeneity in patterns rather than to maximize between-group statistical significance on individual variables [28,31]. Even when differences in specific functions are not statistically significant, the cluster solution may still provide meaningful insights.

First, the overall profiles of the clusters reflect distinct combinations of lifestyle and technology needs that are not captured when examining single variables in isolation. Prior work has emphasized that segmentation should be evaluated on interpretability and theoretical coherence, rather than significance tests alone [29,32]. Second, the relatively small sample size in some clusters may have limited statistical power to detect differences. This limitation is particularly relevant for cluster 2, but the stability of this subgroup across repeated clustering iterations supports its substantive validity.

Thus, while statistical tests provide one form of validation, the meaningful differentiation in the patterns of technology needs across clusters—combined with theoretical plausibility and methodological robustness—justifies the retention and interpretation of the 4-cluster solution. Comparing these results with the segmentation and smart home needs analyzed previously in the Korean study [11], we noticed that the median value of smart home needs in South Korea was 3 (IQR 1-4), which is higher than in Spain (2, IQR 1-2). We believe that the difference in the popularization of smart homes in the 2 countries [33,34] and the internet use among the older adults can affect overall smart home perception and needs. It is possible to establish some parallels between clusters in Spain and in Korea. For instance, cluster 1 “high quality of life and independent life” in Spain and Korea’s “high quality of life and healthy” have similar RBL and had the lowest demands for smart home functions compared to other groups. However, we can highlight that although Spain and Korea have similarities, the segmentation extracted different clusters and needs. This difference arises because instead of imposing a top-down classification, we obtained the clusters in a bottom-up fashion, maintaining in this way the particularities of each country. We believe this is a very useful tool to understand the idiosyncrasy of each culture and to better design smart home technologies that satisfy older adults’ needs.

### Limitations

A first limitation can be pointed out regarding the number and heterogeneity of participants: Due to the characteristics of the non–face-to-face survey, we could not target all the older adults in Spain but only those older adults who could respond to an internet survey. Also, the number of responses collected is related to unfamiliarity with online surveys for the older adults in this country. However, the use of information and communication technologies is increasing year by year in Spain. In particular, the daily internet usage rate among those aged 65 to 74 years increased to 50.7% in 2020, compared to 43.3% in 2019 [35,35]. We believe it is a practical method to quickly gather data and reach conclusions for the older population, and we are confident that it will become a more effective method in Spain.

The channel for the distribution of the survey may also have an impact on the educational level of respondents. We collected 78.43% of responses from older people with university education, which is significantly higher than the percentage of Spanish adults who have reached tertiary education (approximately 38%). In addition, because participants were able to complete this online survey alone, we acknowledge there could be a bias in our sample to older adults with good cognitive status, familiarity with technology, and possibly a higher willingness to adopt smart home technologies. This can pose a threat to the representativeness of the sample, which is important, as smart home technology could especially benefit older adults with cognitive decline. In future research, we aim to collect feedback from older adults with lower educational levels and more varied cognitive status, to increase the representativeness of the results. Further, given that the survey was anonymous, and we were not controlling who the person answering the questionnaire was, there was a risk of collecting responses from participants who do not meet the target profile. However, the channels that we used for the distribution of the questionnaire increase our confidence in effectively being able to reach the target population.

Another issue to be highlighted is the low number of respondents who were classified into cluster 2 “poor quality of life” (n=5). As a result, the demographic characteristics and responses of each individual member have a considerable influence in the definition of the cluster. We were concerned about the small dimension of cluster 2, so we tested grouping in various ways, but these 5 adults could not be tied to other groups. Although the cluster with 5 people was a small group, we think it is a meaningful group because of their distinct characteristics and RBL. In addition, small but stable clusters have been shown in prior literature to capture unique patterns or extreme profiles that larger clusters cannot reveal [29]. Retaining this cluster therefore enhances our ability to reflect the full heterogeneity of the data, even if statistical generalizations must be made with caution. Following established recommendations [36], we decided to keep the 4-cluster solution, while explicitly noting the interpretive limitations associated with the small sample size of cluster 2.

We believe the reduced number of participants in this group may be due to limited internet access of older people with this profile. We observe that the proportion of members aged older than 75 years in this cluster is the highest (20%), whereas the representation of this age segment in the other 3 clusters is much lower (with a maximum of 8%). Considering that the quality of life tends to decrease with age, this cluster might be biased to be defined by the low quality of life of its members. We would like to explore this issue by collecting more responses on the older age segment.

A more fundamental limitation of this study (but also one of its strengths) is that the results are inherently dependent on the target population. Spanish RBLs may differ from those in other countries and cultures. Being a data-driven research process, the factors that emerge through PCA are different, and the clustering process also leads to groups that are not necessarily comparable to the ones in another population. Direct generalization of the conclusions to another population should therefore be avoided. However, we consider this as a major strength of our method. The independent results of the segmentation by countries (Spain and South Korea) are evidence that smart home technology must be tailored to each specific context. This method may help to investigate the context and guide the tailoring process in technology development and deployment. This is also an interesting tool to guide policymakers in deciding concrete actuations to foster the integration of smart home technologies with existing services.

### Conclusions

A smart home is an assistive technology that can improve the quality of life in old age and lead to an independent life. In order for this rapidly growing technology to fulfill its role in the lives of older people, it is necessary to first identify and analyze the actual needs of potential users. Older people are generally going through stages that may be physically and mentally debilitating, but their lifestyles at home cannot be defined as homogeneous. If the specific needs of older people are met by analyzing and segmenting life patterns at home, this assistive technology can fully perform its role. Our main contribution is a method that can be applied to any older adult group to analyze their smart home needs. In this study, we applied previously defined methods and questionnaires in a different geographical context. As confirmed through the analysis results, there was a difference in the priority of smart home functions and the number of functions required according to segmentation by RBL. It was also confirmed that RBL-based smart home needs analysis is meaningful even for studies conducted in different countries with different cultures. This second study demonstrates that the proposed questionnaire and research method are generally useful and not limited to specific countries or contexts. The results of this study and the previous study in South Korea showed that this method can be used to investigate the perception of smart home technology in older adults and identify the actual needs of the groups that emerge.

Future research may explore a more sophisticated yet comprehensive categorization of smart home features, exploring the possibility of hierarchically grouping the 26 individual smart home functions into function categories or themes, to improve the interpretation of the preferences of different user groups.

## Abbreviations

MCS: mental component summary
PCA: principal component analysis
PCS: physical component summary
RBL: residentially based lifestyles
